# The effects of a group cognitive behavioral therapy program using video communication for pregnant women with depressed mood in Korea: a pilot study

**DOI:** 10.4069/kjwhn.2021.11.15

**Published:** 2021-12-15

**Authors:** Eunjoo Lee, Mijung Kim

**Affiliations:** 1 Department of Nursing, Kyungnam University, Changwon, Korea; 2 Department of Nursing, Masan University, Changwon, Korea

**Keywords:** Cognitive behavioral therapy, Depression, Pregnant women, Republic of Korea, Self report

## Abstract

**Purpose:**

This study presents the development of a group video communication-based cognitive behavioral therapy (CBT) program for depressed pregnant women. It also provides the results of a preliminary test of its effects on their depression, automatic thoughts, and dysfunctional attitudes.

**Methods:**

In this quasi-experimental single-group pre- and posttest design study, 13 pregnant women participated in a 4-week, eight-session group CBT program, based on Beck’s cognitive theory and using video communications from November 2020 to January. Pregnant women between 14 and 32 weeks who were members of an online maternity and parenting community and residing in the cities of Changwon and Gimhae, Korea, were invited to voluntarily participate. Trained nurses led CBT sessions of 3–4 participants per group via video communication. Participants were assessed pre- and postintervention with self-report questionnaires for measurement of depression, automatic thought, and dysfunctional attitude after normality test according to the Shapiro-Wilk test of the variables. The data were analyzed using paired t-test and Pearson correlation coefficients.

**Results:**

Depression (t=7.90, *p*<.001), automatic thoughts (t=4.89, *p*<.001), and dysfunctional attitudes (t=2.42, *p*=.032) significantly decreased after the 4-week online program. There were statistically significant correlations among the three variables.

**Conclusion:**

This program was found to be effective in reducing depression, automatic thoughts, and dysfunctional attitudes. Above findings suggest that a group CBT program using video communication can be an effective therapeutic modality that helps pregnant women at risk for depression alleviate their negative emotions related to depression.

## Introduction

Perinatal depression, which encompasses severe or mild depressive episodes that occur during pregnancy and postpartum, is one of the most common medical complications [1]. It is reported to occur in 1 in 7 women worldwide [1] and thoughts of intentional harm to themselves or their babies accompany the typical symptoms of depression [2]. Perinatal depression is regarded as a major public health challenge because if left untreated, it can incur major losses and expenses for not only the affected women but also their families and the communities [3].

Since perinatal depression occurs during pregnancy or the first 12 months after delivery [1] and postpartum depression (PPD) develops during or after pregnancy [4], early management of depression during pregnancy is of the utmost importance. To detect depression in its early stages, pregnant women are recommended to receive screening for depression throughout pregnancy, and those who are at high risk for depression or experience suicidal thoughts are closely monitored [1]. However, even women who receive scores exceeding the threshold for depression in a screening test often do not receive active treatment due to the lack of time, limited access to medical services (in terms of time, location, and transportation), stigma related to psychiatric treatment, and a lack of awareness of the need for mental health management [4].

Individuals vulnerable to depression have dysfunctional attitudes toward themselves, the world, and the future, and they can have automatic thoughts and cognitive errors [5]. Dysfunctional attitudes and irrational beliefs based on past experiences lead to the deployment of selective attention to external stimuli [5] and distorted patterns of responses to feedback [6]. Automatic thoughts refer to a pattern of thinking that recurs in connection with specific situations and is activated by dysfunctional beliefs [5]. In a qualitative study on the experience of depression, pregnant women had distorted perceptions of reality in relation to childhood experiences or negative situations, and they lost control of their emotions due to the recurrence of negative thoughts [7].

Cognitive behavioral therapy (CBT) is effective for changing irrational, distorted thoughts into rational thoughts and reducing negative automatic thoughts [8]. It is also a type of psychotherapy that helps people learn to understand and correct destructive and distorted thinking patterns that negatively affect emotions and behaviors [9]. According to Beck’s cognitive theory [10], since an individual’s thinking or cognition determines his or her emotions and behaviors, a cognitive approach to cognitive biases or erroneous beliefs can achieve new adaptive thoughts and behavior changes. In other words, CBT can be expected to bring about changes in emotions and behaviors through changes in negative thinking, such as dysfunctional attitudes and automatic thoughts. Changes in thoughts shift individuals’ core beliefs and dysfunctional beliefs derived from automatic thoughts, thereby playing an important role in preventing depression [11]. Likewise, depression, automatic thoughts, and dysfunctional attitudes are closely related, making it necessary to verify them as variables affected by CBT aiming to reduce depression.

In CBT, feedback and communication between the therapist and the client are important; thus, the coronavirus disease 2019 (COVID-19) pandemic has made the application of face-to-face CBT difficult. However, online CBT is advantageous insofar as it enables interventions involving interactions between the therapist and the client without restrictions on time and place [12]. Compared to individual treatment, group CBT is more efficient in terms of time and cost, since it provides the benefits of treatment to many people [12] and is effective at alleviating negative psychological effects such as depression, automatic thoughts [8], emotion regulation [9], and anxiety [12]. Furthermore, the ability of video communication to enable real-time communication among users and immediate feedback is expected to augment the effect of therapeutic interventions in CBT.

Previous international studies have demonstrated that CBT reduces depression in pregnant women [13-15] and that online CBT is effective [16,17]. In South Korea, however, there is a paucity of research on interventions using CBT, although there are reports on prenatal education programs with spouse participation [18] and PPD counseling programs for husbands [19].

This study aimed to develop a video communication-based group CBT program to reduce depression during pregnancy and to verify its effectiveness. The specific objectives were as follows. First, to develop a video communication-based group CBT program for pregnant women with depressed mood. Second, to investigate the effectiveness of the video communication-based group CBT program by applying it to depressed pregnant women. Third, to identify the correlations between automatic thoughts, dysfunctional attitudes, and depression in depressed pregnant women.

This study had the following hypotheses.

• Hypothesis 1. There will be differences in depression among pregnant women before and after participating in the video communication-based group CBT program.

• Hypothesis 2. There will be differences in the automatic thoughts of pregnant women before and after participating in the video communication-based group CBT program.

• Hypothesis 3. There will be differences in dysfunctional attitudes of pregnant women before and after participating in the video communication-based group CBT program.

• Hypothesis 4. There will be significant correlations between automatic thoughts, dysfunctional attitudes, and depression in depressed pregnant women.

## Methods

Ethical statement: This study was approved by the Institutional Review Board of Kyungnam University (No. 1040460-A-2020-039). Informed consent was obtained from the participants.

### Study design

This quasi-experimental single-group pre-experimental study was designed to develop a video communication-based group CBT program for pregnant women with depression and to evaluate its effectiveness. This study report followed the TREND (Transparent Reporting of Evaluations with Nonrandomized Designs) reporting guidelines [20].

### Participants

The participants in this study comprised members of an online community focused on maternity and parenting (“Mom Café”), who resided in the cities of Changwon and Gimhae in Gyeongsangnam-do Province, South Korea. The specific inclusion criteria were as follows: 1) 20 years of age or older, 2) between 14 and 32 weeks of pregnancy, 3) a PPD test (Edinburgh Postnatal Depression Scale, EPDS) [21] score of 9 or higher, 4) the ability to understand and respond to the questionnaire, 5) owning computers or smartphones capable of video communication, and 6) voluntary participation in this study. The exclusion criteria were 1) being diagnosed with a mental illness and taking drugs or receiving psychiatric treatment, 2) having serious pregnancy complications, 3) non-Korean citizenship, and 4) illiteracy. The cut point for the EPDS score was selected as 9, corresponding to the level that requires counseling according to the recommendations of the Ministry of Health and Welfare and the Korea Disease Control and Prevention Agency (KDCA) [22] (0–8 points, normal; 9–12 points, borderline [counseling required]; ≥13 points, severe [treatment required]).

We posted a study enrollment notice on Mom Café and asked those who were willing to participate in the study to call the researchers by phone. A file to measure depression was sent through social media to those who agreed to voluntarily participate in the study after receiving explanations of the purpose and process of the study. Of these individuals, those with a score of 9 or higher were eligible to participate in the study. We also explained to the participants that they could withdraw from the study at any time, that the collected data would not be used for purposes other than the research, and that the confidentiality of personal information would be guaranteed. It was explained that if a physical or psychological problem occurred in a participant during the program, study participation would be immediately discontinued for the individual to rest and to be transferred promptly to a medical institution to receive appropriate treatment.

#### Study size

The minimum number of participants required in a single group was 12 when estimated using G*Power version 3.1.9 with a significance level of .05, an effect size of .80, and a power of .80 with a one-sided test. In previous Korean experimental studies involving pregnant women [12,19], the number of participants was around 10. Due to the low participation during the prolonged COVID-19 pandemic, a total of 13 people who agreed to participate in the study were included in the final analysis.

### Measurement

#### Depression

Depression was assessed using the Korean version EPDS scale, which is posted on the National Health Information Portal of the KDCA [22]. The reliability and validity of this tool have been demonstrated worldwide, and the EPDS is considered a representative tool for evaluating depression that occurs during the perinatal period (during pregnancy and postpartum) [4]. The 10 items consists of several items dealing with topics such as anxiety, worry, fear, miserable feelings, unhappy feelings, and self-harm ideation experienced by an individual during the past week. (i.e., not at the time of the test). A higher total score (range: 0–30 points) indicates a higher degree of depression. Internal reliability was good at the time of tool development [21], Cronbach’s α of .85, as well as in the current study (Cronbach’s α of .84 before the intervention and .83 afterwards).

#### Automatic thoughts

To assess automatic thoughts, we used the Korean version of the Automatic Thoughts Questionnaire-Negative [23] adapted by Kwon and Yun [24] after obtaining their permission. This tool was designed to evaluate an individual’s level of negative thoughts in daily life and has subdomains including dissatisfaction with reality and desire for change, helplessness and loss of motivation, and negative thoughts on one’s own life and negative expectations. It consists of a total of 30 items scored using a 5-point Likert scale (‘not at all,’ 1 to ‘all the time,’ 5). A higher total score (range: 30–150) indicates a higher degree of automatic thinking. Cronbach’s α was .94 when previously used in Korea [24]; in the current study, it was .92 before the intervention and .94 after the intervention.

#### Dysfunctional attitudes

For dysfunctional attitudes, a tool adapted by Kwon [25] from the dysfunctional attitudes scale [26] was used with permission from the authors. This 40-item tool describes common maladaptive beliefs among depressed patients. The summed scores (range, 40–280) are rated on a 7-point Likert scale (‘strongly disagree,’ 1 to ‘strongly agree,’ 7) and higher scores indicate a higher level of dysfunctional attitude. Internal reliability was stable in Kwon’s study [25], Cronbach’s α was .86. In the current study, Cronbach’s α was .95 before the intervention and .88 after the intervention.

#### General and obstetric characteristics

General and obstetric characteristics included age, education, occupation, monthly income, gestational age (weeks), number of children, complications of pregnancy, planned pregnancy, and family history of depression.

### Setting and data collection

This study was conducted from November 23, 2020 to January 30, 2021, and the detailed research process was as follows.

#### Development of the video communication-based group CBT program to reduce depression in pregnant women

A draft of the program was constructed based on Beck’s cognitive theory [10], referring to the literature [6-9] related to perinatal depression and the results of small-group exploratory interviews. The small-group exploratory interviews using a video communication platform (Zoom; Zoom Video Communications, Inc., San Jose, CA, USA) were conducted with five pregnant women who had a high level of depression (an EPDS score of 9 or higher) to confirm their needs for the program. During the interview, the following open-ended questions were asked: “What are the symptoms of depression experienced during pregnancy?,” “How are you managing depression?,” “How do you get information to prevent depression?,” “What methods do you use when depressed?,” “Are there any depression management methods that you would like to know more about?,” “If you do not manage your depression, what is the reason?,” “What would you like to learn from this program?” The interview content was recorded on Zoom and then transcribed to analyze participants’ needs. Based on these interviews, we tried to investigate how to recognize depression, prevent depression in pregnant women, locate the causes of depression, and resolve stress, depression, and negative thoughts.

The CBT model was designed to apply adaptive conscious thinking such as rational thinking and problem-solving by identifying negative automatic thoughts and core beliefs with a focus on the relationship between individual thoughts, emotions, and behaviors [6]. In previous studies, women who experienced depression during pregnancy were stressed about pregnancy-related situations, often did not know whether they were depressed, and perceived the situation negatively [7]. Therefore, cognitive reconstruction in this program was conducted to explore automatic thoughts and core beliefs related to pregnancy and modify them into alternative thoughts. For this purpose, we used identification of stress, self-reflection, exploration of cognitive errors, location of solutions, emotion control training, and exploration of methods to resolve stress related to pregnancy and child care. The program was evaluated for content validity by three experts, including one obstetrician-gynecologist, one psychiatric nursing professor, and one CBT specialist. According to the opinions of the experts, sessions were organized to reflect the unique characteristics of pregnant women, and activities aimed at enhancing interest in participation in the program and improving focus were added.

The program involved eight sessions (twice a week for 4 weeks) based on previous studies [12,18] and is presented in Table 1. Each session was 80 minutes, with CBT content consisting of 50 minutes and 30 minutes for introduction, overview, and checking on assignments. In the first session, introductions were made, program rules were set, and education on cognitive behavior and a PPD summary were provided. The second session aimed to help participants understand their emotions and thoughts, by finding emotional words (pleasant words and unpleasant words) that they use frequently and to recall their emotions, actions, and subsequent positive and negative situations. In the third session, a drawing activity using a projection technique was conducted to improve their understanding of how to identify stress, and they were asked to think about their thoughts and actions in conflict-inducing situations. The fourth session focused on reflecting on their core beliefs and problem-solving in stressful and conflicting situations during pregnancy and parenting and sharing experiences of depression during pregnancy. In the fifth session, participants were encouraged to recall positive and negative experiences of pregnancy and find their own cognitive errors in conflict situations during pregnancy. In the sixth session, animals that could best describe their shaken emotions were discussed and classification of negative automatic thoughts, core beliefs, and cognitive errors was done. In the seventh session, participants examined their emotions, thoughts and actions, and problem-solving methods in conflict situations. In the final session, the participants compared their emotions, thoughts, and behaviors in conflict situations before and after the program and were asked to reflect on their changes.

#### Pre- and postintervention assessments

The questionnaire was sent via email or social media (KakaoTalk; Kakao Corp., Jeju, Korea) before the start of the session and immediately after the end of the eighth session. It took about 10 to 15 minutes to fill out the questionnaire, and the participants were provided with a gift in return for their participation in the study.

#### Intervention

The 13 participants were divided into four experimental groups by assigning three to four participants to each group on a first-come, first-served basis. Each group participated in eight sessions of the group CBT program (twice a week) for 80 minutes. A research assistant contacted the participants in advance before the start of each session to check whether they were connected for video communication and to help them connect smoothly. The session was conducted in the following sequence: sharing life experiences after the last session, sharing the main learning content of each session and feelings about the activities, and instructions on the assignment. At the end of the session, the assignment was explained and participants were asked to send their finished assignment before the next session via email or social media. Assignments were given in stages according to the progress of the session, and the researchers checked whether the group members understood and achieved the activity goals and gave additional individual feedback when explanations were necessary. The submitted assignments were shared with other members of the group during group activities in the next session. Assuming that self-disclosure might be difficult due to prejudice against people with depression, the participants were asked to select an alias during the first self-introduction session, and it was used until the end of the program.

A researcher and a research assistant operated the program for each group. The lead researcher was a professor of women’s health and nursing who had been conducting research related to perinatal depression for more than 10 years. The research assistant had worked as a mental health nurse in the psychiatric department and currently runs a program that applies CBT techniques to people with depression, high risk of suicide, and psychiatric disorders at a mental health welfare center.

### Data analysis

The collected data were analyzed using IBM SPSS for Windows ver. 23.0 (IBM Corp., Armonk, NY, USA). Descriptive statistics were done for demographic characteristics and preintervention dependent variables (depression, automatic thoughts, and dysfunctional attitudes). The relationships between automatic thoughts, dysfunctional attitudes, and depression were analyzed with Pearson correlation coefficients. The Shapiro-Wilk test was performed to verify the normality of the dependent variables; all variables showed a normal distribution, so the differences between the pre- and postintervention measurements were analyzed using the paired t-test.

## Results

### General and obstetric characteristics

The average age of the participants was 33.46±2.40 years and ranged from 30 to 37 years. Eight of the participants (61.5%) were under the age of 35 years (the threshold used to define advanced maternal age). Six participants (46.2%) had a community college degree, seven participants (53.8%) were employed, and the most common (46.2%) monthly income level was between 3 million to 4 million Korean won (roughly 2,700–3,600 US dollars), which is similar to the national average.

The average number of pregnancies was 1.85±0.80, and the mean gestational age was 17.15±2.73 weeks, ranging from 14 to 24 weeks. Nine participants (69.2%) were in the second trimester (15–28 weeks), and of the seven participants (53.8%) who already had children; most of them (n=6, 85.7%) had one child. Although one participant had pregnancy-related hypertension, it was a mild stage. Ten (76.9%) had a planned pregnancy, and two (15.4%) had a family history of depression (Table 2).

### Relationship between automatic thoughts, dysfunctional attitudes, and depression

Depression had a significant positive correlation with automatic thoughts (r=.79, *p*<.001) and a significant positive correlation with dysfunctional attitudes (r=.63, *p*=.020). Dysfunctional attitudes showed a significant positive correlation with automatic thoughts (r=.56, *p*=.045) (Table 3).

### Verification of the effect of the video communication-based group CBT program

Compared to before the intervention. Depression (t=7.90, *p*<.001), automatic thoughts (t=4.89, *p*<.001), and dysfunctional attitudes (t=2.42, *p*=.032) significantly decreased after the intervention (Table 4).

## Discussion

The purpose of this study was to develop a video communication-based group CBT program for reducing depression in pregnant women and to verify the effects of the program on depression, automatic thoughts, and dysfunctional attitudes after participation in the program. Referring to the elements of cognitive therapy, we designed the program to have the following therapeutic elements: exploration of automatic thoughts and core beliefs in daily life and pregnancy situations, identification of stress, self-reflection, emotion control training, and exploration of problem-solving methods.

Depression significantly decreased after the intervention. Although a direct comparison is difficult due to the absence of previous studies including a single group, a comparable finding was reported in a study where CBT treatment improved depressive symptoms in pregnant women more effectively than conventional treatment [13]. In an overseas study that applied a 1-hour online video CBT for 13 weeks, patients’ depression level was significantly reduced from moderate to low [27]. Real-time CBT can be expected to effectively reduce depression by identifying unrealistic or erroneous expectations among depressed subjects and by managing their expectations through explorations of possible problems that can occur if expectations are not managed [28]. This study’s decrease in depression score from moderate to low after the intervention may be related to participants developing the will to resolve depression on their own by recognizing their emotions and thoughts and confronting depression through the program. Our interpretation is supported by the results of Kwon and Lee [11], who reported that depression could be reduced through CBT, which changed individuals’ negative thinking systems by enabling them to examine and verify their thoughts. Since both cognition and behavior are closely related to mood [2], if it is possible to separate emotions through CBT, depressive emotions could be reduced by detecting errors and changing thoughts. In particular, it was possible to establish a good cooperative relationship for treatment through real-time video communication [28], and a possible reason why depression was reduced may have been that the participants felt empathy and comfort while sharing their pain and conflicts related to pregnancy. Early detection and treatment of depression during pregnancy are vital to prevent PPD [2]. Screening pregnant women at high risk of depression and providing them with a video communication CBT program could reduce depression during pregnancy, thereby preventing a transition to PPD. An EPDS tool effective for screening PPD should be used at antenatal visits to keep the level of depression in pregnant women in check, and CBT experts should be systematically trained and dispatched to adjacent obstetrics and gynecology clinics. In addition, it is necessary to encourage social awareness that depressive symptoms can frequently occur in pregnant women, that treatment is possible, and that pregnant women should seek out treatment for depression if necessary.

Automatic thoughts were significantly reduced after the intervention using the video communication-based group CBT program compared to before the intervention. In a prior study of low-income children and adolescents [8], a CBT program also reduced negative automatic thoughts, and the effect remained present at 8 weeks after the intervention. Examining cognitive errors reflected in automatic thoughts is a very effective way to change automatic thoughts [29], which makes it possible to achieve the ultimate goal of CBT for depression. In order to reduce automatic thoughts, we designed our program to include exercises for locating cognitive errors by exploring the automatic thoughts felt in negative situations and for changing thoughts and emotions in a stepwise manner. In addition, video communication may have facilitated the detection of automatic thoughts and errors through the interactive exchange of opinions or feedback between the researchers and the participants.

Dysfunctional attitudes were significantly reduced after the intervention, which in is line with a prior study testing CBT for adolescents by Jeong and Kim [29], dysfunctional attitude scores significantly decreased after the intervention. People with depressive disorder have negative views about incidents in daily life, and the purpose of treatment for depression in CBT is to help clients process information in a rational way [6]. This change in dysfunctional beliefs helps people better cope with negative life incidents, thereby lowering the recurrence of depression even after treatment [25]. However, in the study of Jo and Son [30] that investigated the effect of a group CBT program for college students (twice a week, 90 minutes per session, for a total of 12 times), there was no significant difference in dysfunctional attitude scores before and after the intervention, contrary to the result of our study. As the latter study [30] CBT program was applied to participants who had job stress scores and dysfunctional attitude scores in the top 25%, the difference in job-related situations among the participants, negative beliefs or their coping methods might have been difficult to identify. In our program participants were more homogeneous, e.g., had depressive mood and were coping with the same pregnancy-related situations, and thus, could share their problem recognition and problem-solving methods. We believe this may have helped them become aware of their own attitudes that interfered with problem-solving when they had difficulty in adjusting to pregnancy and dealing with uncomfortable situations, thereby reducing dysfunctional attitudes.

Depression showed a significant positive strong correlation with automatic thoughts and dysfunctional attitudes. Higher levels of depression were associated with higher levels of automatic thoughts and dysfunctional attitudes, which are factors that influence the onset of depression [11]. In addition, negative thoughts are related to depressive mood and act as a vulnerability factor for the onset of depression [11]; therefore, negative thoughts are considered to have a proportional relationship with depression. Dysfunctional attitudes showed a significant positive correlation with automatic thoughts. Since automatic thinking is one of the characteristics of dysfunctional thinking [11], the close correlation is logical. However, since a moderate or higher correlation was noted for automatic thoughts and dysfunctional attitudes, it may be difficult to isolate the effect of each variable completely, undermining the accuracy of the results.

The main limitation of this study is that it is difficult to generalize its results, since the study contained only an experimental group, without a control group, and the sample size was small due to the difficulty of recruiting study participants. In addition, although participants were at different stages of pregnancy, it was not possible to investigate aspects of negative psychology according to pregnancy stage; and in some cases online access for video communication was not smooth. However, as a preliminary study, it offers information for utilizing a group video communication CBT program for pregnant women with depressed mood in the future. Strategic planning for targeted recruitment, and ensuring online connection may be needed. Furthermore, to confirm the lasting effects of the program, future research needs to consider longitudinal changes in depression and the characteristics of each pregnancy period.

In conclusion, this nurse-led eight-session group CBT program for pregnant women with depressed mood via video communication appeared to be helpful in reducing levels of depression, automatic thought, and dysfunctional attitude after 4 weeks. The significant changes in main variables noted after the program may be attributed to the formation of trust between the therapist and group members through video communication, which facilitated self-disclosure. This pilot study also it demonstrated the feasibility and applicability of an online-based group CBT program format, in that the participants were able to reveal their faces to other pregnant peers and immerse themselves in the program. The group activities involving pregnancy context helped the participants obtain empathy and comfort from other participants who were in the same situation, which may have buffered their negative psychology about pregnancy and childbirth. This study has potential for alleviating prenatal depression and preventing PPD, especially when activities for pregnant women are socially limited, e.g., due to the ongoing COVID-19 pandemic, and should be further examined in future studies.

## Figures and Tables

**Table 1.** Content of the group cognitive behavioral therapy program using video communication

Session	Subjects	Contents	Group activities
1	Program introduction	• Introduction to the program course	20 minutes
	Cognitive behavior concept	• Cognitive behavior concept	Introduction to group members
	Building relationships	• Postpartum depression concept	Establishing group rules
		• Group activities	
		• Setting activity goals and expectations	
		• Assignment (setting priorities in everyday life)	
2	Understanding one’s own emotions	• Reviewing last week’s life experiences and homework	50 minutes
		• Identifying own thoughts, feelings, and actions	Sharing emotions and behaviors in negative situation
		• Group activities	
		• Assignment (examine one’s own thoughts and feelings)	
3	Cognitive vulnerability	• Reviewing last week’s life experiences and homework	50 minutes
	- stress perception	• Checking daily life stress	Checking stress through painting
		• Exploring core beliefs and solutions related to conflict	Sharing how to solve the problem
		• Group activities	
		• Assignment (checking for negative thoughts and emotions)	
4	Cognitive vulnerability	• Reviewing last week’s life experiences and homework	50 minutes
	- cognition of pregnancy stress	• Checking pregnancy and parenting stress	Sharing how to deal with pregnancy and parenting stress
		• Explore core beliefs and solutions related to conflict during pregnancy	
		• Group activities	
		• Assignment (checking for negative thoughts and emotions)	
5	Cognitive reconstruction I	• Reviewing last week’s life experiences and homework	50 minutes
		• Finding one’s own core beliefs	Sharing own cognitive errors
		• Revising one’s own core beliefs	
		• Group activities	
		• Assignment (checking for cognitive errors and altered behavior)	
6	Cognitive reconstruction Ⅱ	• Reviewing last week’s life experiences and homework	50 minutes
		• Distinguishing between automatic thoughts, core beliefs, cognitive errors, and emotions in conflict situations	Sharing experiences of correcting negative thinking
		• Finding one’s own negative automatic thoughts	
		• Group activities	
		• Assignment (comparison of thoughts, emotions, and behaviors before and after pregnancy)	
7	Coping behavior	• Reviewing last week’s life experiences and homework	50 minutes
		• Rehearsing actions	Sharing of changing experiences of problem-solving methods
		• Group activities	
		• Assignment (comparison of thoughts, emotions, and behaviors before and after pregnancy)	
8	Plan of cognitive action	• Reviewing last week’s life experiences and homework	50 minutes
		• Checking the changed self (thinking-feeling-action)	Share rational changes in the future
		• Making a specific action plan	
		• Group activities	
		• Final summary and feedback	

**Table 2.** General and obstetric characteristics of participants (N=13)

Variable	Categories	Mean±SD or n (%)
Age (year)		33.46±2.40
		(range, 30–37)
	<35	8 (61.5)
	≥35	5 (38.5)
Education	College or less	6 (46.2)
	University or higher	7 (53.8)
Occupation	Yes	7 (53.8)
	No	5 (38.5)
	Maternity leave	1 (7.7)
Monthly income (Korean won)*	≤2 million	1 (7.7)
	2.1-3 million	4 (30.8)
	3.1-4 million	6 (46.2)
	4.1 million	2 (15.4)
Frequency of pregnancy		1.85±0.80
Gestational age (week)		17.15±2.73
		(range, 14–24)
	1st trimester (≤14)	4 (30.8)
	2nd trimester (15–28)	9 (69.2)
Presence of older children	Yes	7 (53.8)
	No	6 (46.2)
Number of children (n=7)	1	6 (85.7)
	2	1 (14.3)
Complication of pregnancy	Yes	1 (7.7)
	No	12 (92.3)
Planned pregnancy	Yes	10 (76.9)
	No	3 (23.1)
Family history of depression	Yes	2 (15.4)
	No	11 (84.6)

* One million Korean won is approximately 900 US dollars.

**Table 3.** Relationships among automatic thoughts, dysfunctional attitudes, and depression (N=13)

Variable	r (*p*)	
	Automatic thoughts	Dysfunctional attitudes
Automatic thoughts	1	
Dysfunctional attitudes	.56 (.045)	1
Depression	.79 (<.001)	.63 (.020)

**Table 4.** Differences in depression, automatic thoughts, and dysfunctional attitudes before and after the intervention (N=13)

Variable	Possible score range	Mean±SD		Mean difference	
		Preintervention	Postintervention	t	*p*
Automatic thoughts	30–150	68.75±15.23	49.46±14.27	4.89	<.001
Dysfunctional attitudes	40–280	152.15±29.50	130.23±21.32	2.42	.032
Depression	0–30	14.38±5.10	6.30±3.79	7.90	<.001
